# Six-Year Incidence of Blindness and Visual Impairment in Kenya: The Nakuru Eye Disease Cohort Study

**DOI:** 10.1167/iovs.16-19835

**Published:** 2016-11

**Authors:** Andrew Bastawrous, Wanjiku Mathenge, Kevin Wing, Hillary Rono, Michael Gichangi, Helen A. Weiss, David Macleod, Allen Foster, Matthew J. Burton, Hannah Kuper

**Affiliations:** 1 International Centre for Eye Health, Clinical Research Department, London School of Hygiene and Tropical Medicine, London, United Kingdom; 2 Rwanda International Institute of Ophthalmology and Dr. Agarwal's Eye Hospital, Kigali, Rwanda; 3 Department of Non-Communicable Disease Epidemiology, London School of Hygiene and Tropical Medicine, London, United Kingdom; 4 Global Health and Populations Group, Wellcome Sanger Institute, Cambridge, United Kingdom; 5 Kitale Eye Unit, Ministry of Health Trans Nzoia County, Kenya; 6 Ministry of Health, Nairobi, Kenya; 7 MRC Tropical Epidemiology Group, Department of Infectious Disease Epidemiology, London School of Hygiene & Tropical Medicine, London, United Kingdom; 8 Moorfields Eye Hospital, London, United Kingdom

**Keywords:** Kenya, Africa, visual impairment, blindness, incidence, cohort, population-based

## Abstract

**Purpose:**

To describe the cumulative 6-year incidence of visual impairment (VI) and blindness in an adult Kenyan population. The Nakuru Posterior Segment Eye Disease Study is a population-based sample of 4414 participants aged ≥50 years, enrolled in 2007–2008. Of these, 2170 (50%) were reexamined in 2013–2014.

**Methods:**

The World Health Organization (WHO) and US definitions were used to calculate presenting visual acuity classifications based on logMAR visual acuity tests at baseline and follow-up. Detailed ophthalmic and anthropometric examinations as well as a questionnaire, which included past medical and ophthalmic history, were used to assess risk factors for study participation and vision loss. Cumulative incidence of VI and blindness, and factors associated with these outcomes, were estimated. Inverse probability weighting was used to adjust for nonparticipation.

**Results:**

Visual acuity measurements were available for 2164 (99.7%) participants. Using WHO definitions, the 6-year cumulative incidence of VI was 11.9% (95%CI [confidence interval]: 10.3–13.8%) and blindness was 1.51% (95%CI: 1.0–2.2%); using the US classification, the cumulative incidence of blindness was 2.70% (95%CI: 1.8–3.2%). Incidence of VI increased strongly with older age, and independently with being diabetic. There are an estimated 21 new cases of VI per year in people aged ≥50 years per 1000 people, of whom 3 are blind. Therefore in Kenya we estimate that there are 92,000 new cases of VI in people aged ≥50 years per year, of whom 11,600 are blind, out of a total population of approximately 4.3 million people aged 50 and above.

**Conclusions:**

The incidence of VI and blindness in this older Kenyan population was considerably higher than in comparable studies worldwide. A continued effort to strengthen the eye health system is necessary to support the growing unmet need in an aging and growing population.

Global estimates based on recent population-based surveys suggest that approximately 191 to 285 million people live with visual impairment (VI; defined as visual acuity of <6/18 or <20/60 in the better eye), of whom 32 to 39 million people are bilaterally blind (visual acuity < 3/60 or < 20/400 in the better eye).^1,2^ Overall, VI is ranked sixth in the global burden of disease in terms of disability-adjusted life-years (DALYs)^3^ and is associated with increased mortality.^4,5^ Despite a reduction in the prevalence of blindness in sub-Saharan Africa over the last two decades, the numbers with VI have risen due to an increase in population and longevity,^6^ though data are sparse.

Longitudinal studies provide the opportunity to investigate the natural history of disease, which is essential to plan health services. However, despite a large body of data globally on the prevalence and causes of eye disease, data on incident visual loss from population-based cohorts are limited, due to prohibitive costs and complex logistical and planning challenges. Consequently, to date, no longitudinal, population-based studies of eye disease have been undertaken in sub-Saharan Africa, and there have been only a small number worldwide, predominantly in high-income settings.^7–10^ Inferring data from high-income cohorts is not appropriate for low-income settings, and data are required from low-income countries for effective planning of eye care services.

The aim of this study was to estimate the 6-year incidence and risk factors for incident VI and blindness (both bilateral and unilateral) in a cohort of adult Kenyans.

## Materials and Methods

The methodology of the Nakuru Eye Disease Cohort Study has been reported in detail previously^11^ and is summarized here.

### Ethical Approval

The study adhered to the tenets of the Declaration of Helsinki and was approved by the Ethics Committee of London School of Hygiene and Tropical Medicine at both baseline and follow-up (LSHTM Ref. 6192). Baseline approval was provided by the Kenya Medical Research Institute Ethics Committee and by the African Medical and Research Foundation (AMREF) Ethics Committee, Kenya, for the follow-up (AMREF-ESRC P44/12). For both phases, approval was granted by the Rift Valley Provincial Medical Officer and the Nakuru District Medical Officer of Health. Approval was sought from the administrative heads in each cluster. Informed written (or thumbprint) consent was obtained from all participants after the objectives of the survey and the examination process were explained to those eligible in the local dialect, in the presence of a witness. Participants identified with eye conditions, or other health conditions, were referred to local services.

### Baseline Study Population

The baseline population-based survey was conducted in 2007–2008. The sample size of 5000 participants aged ≥50 years was calculated based on an expected prevalence of visual acuity (VA) < 20/40 in the better eye due to posterior segment eye diseases (PSED, the primary outcome for the baseline survey) of 3.0% in this age group, precision of 0.5%, design effect of 1.5, and a response rate of 90%.

A total of 100 clusters each of 50 participants were selected with a probability proportional to the size of the population across Nakuru district. Households were selected within clusters using a modified compact segment sampling method.^12^ An eligible individual was defined as someone aged ≥50 years living in the household for at least 3 months in the previous year. All participants were invited to undergo a comprehensive ophthalmic examination at a screening clinic (details below).

In total, 4381 (response rate 87.4%) participants underwent complete (ophthalmic and general) examination at baseline. Among those aged ≥50 years at baseline, the prevalence of blindness was 1.6% (95%CI [confidence interval]: 1.2–2.1%), and prevalence of VI was 13.6% (95%CI: 11.8–16.0%).^13^

### Follow-up

Follow-up was conducted from January 2013 to March 2014.

#### Retracing at Follow-up: Advance Team.

One week before the follow-up examination clinic was planned for a given cluster, a field officer studied the maps of the village including Global Positioning System (GPS) coordinates recorded at baseline and made phone contact with the village chief or guide to arrange the visit. At the planning visit, a list of study participants was given to the chief, and a local village guide was recruited to assist location of the study participants. At this visit, the examination site was established. Two days prior to the clinic, the field officer reminded chiefs of the visit by phone and notified them and the guide of the advance team's arrival.

On the day prior to the examination clinic, the advance team visited homes of baseline participants, confirmed their identity using National Identity cards, and invited them to attend the examination clinic the following day. All identified participants were also asked to help locate baseline participants who had not been found.

#### Examination Clinic.

The following procedures were undertaken for all participants who attended the examination clinic at both baseline and follow-up, and further details are available elsewhere.^11^ Procedures undertaken but not included in these analyses are not described in this report (e.g., visual field assessment).

##### Registration.

On the examination day, the advance team confirmed the identity of participants against data from baseline (age, date of birth, name, and identity cards). In cases of uncertain identity, confirmation was made based on retinal examination verified by comparison of retinal photos with baseline photo (*n* = 12).

##### Visual Acuity Assessment.

Presenting VA was measured using a back-illuminated modified logMAR reduced tumbling E chart (Sussex Vision, Inc., Rustington, UK),^14,15^ which has been used in previous population-based studies.^16,17^ Presenting VA was measured on all participants, that is, the patient's own correction was used if normally worn.

If the subject's vision was too poor to read any letters on the chart at 4 m, the subject was tested at 1 m, then as follows:

- Counting fingers (CF): ability to count fingers at 1-, 2-, or 3-m distance
- Hand motion (HM): ability to distinguish if a hand is moving or not in front of the patient's face
- Light perception (LP): ability to perceive any light
- No light perception (NLP): inability to see any light or total blindness

Those who did not read 24 letters (VA < 20/40) at 4 m were scheduled for correction and to undergo a repeat VA measurement with the correction in place unless the vision was worse than CF, in which case no correction was undertaken.

##### Anthropometry

A nurse performed and recorded measures of participants: height (Leicester Height Measure; Chasmors Ltd, London, UK); weight (Seca 761 Medical Class 4 Scales mechanical ground scale; Williams Medical Supplies, London, UK); waist and hip circumference (Chasmors Ltd WM02 Body Tape measure); and three measures of blood pressure (Omron Digital Automatic Blood Pressure Monitor Model HEM907; Omron, Hoofddorp, The Netherlands), each 10 minutes apart. In addition, at follow-up, bioimpedence (Tanita Segmental Body Composition Monitor; Tanita, Amsterdam, The Netherlands) was performed.

At baseline, capillary blood was taken from all participants for random blood glucose. Random blood glucose was also taken at follow-up with the addition of glycosylated hemoglobin (HbA1c) in all with a self-reported history of diabetes or random blood glucose of ≥7.0 mM, and a further 10% of nondiabetics (based on history and random blood glucose).

#### Interview.

An interviewer performed a structured interview in the participant's preferred language covering demographic details; past medical and ocular history; known risk factors (e.g., smoking and tobacco consumption and alcohol intake); and socioeconomic status (e.g., job, housing conditions, ownership of material goods and livestock).^18^

### Definitions and Statistical Analyses

All participants who had complete examinations at baseline who were not visually impaired or blind were considered “at risk” for incident VI or blindness, respectively. Follow-up status at 6 years was categorized as Found and examined; Found and not examined; Deceased; Moved away; or Unknown.

Statistical analysis was performed using STATA v13 (Stata Corp, College Station, TX, USA). All analysis accounted for the cluster survey design using Taylor linearized variance estimation to calculate standard errors.

### Preparation of Cohort for Analysis

Pearson χ^2^ tests corrected for the survey design were used to calculate *P* values in order to assess differences between participants seen and those lost to follow-up (LTFU), and between those known to have died and with unknown outcome status. *P* < 0.1 was considered to represent a statistically significant difference.

Those who were deceased were then excluded. Those followed up but without complete records for all covariates at baseline were also excluded.

An inverse probability weighting (IPW) model^19^ was developed to allow estimation of cumulative incidence while accounting for those LTFU. Multivariable logistic regression was used to identify independent baseline covariates associated with LTFU. Covariates for which there was evidence of univariable association with the outcome (*P* < 0.1) were kept in a multivariable model. From this final model, the probability of being followed was estimated, based on the presence or absence of each of these baseline covariates. The inverse of this probability formed the weighting to be applied in order to account for those LTFU.

The final step was to remove those individuals LTFU from the cohort, so that all subsequent analysis would be performed on only those with complete outcome records, with IPW applied to account for those LTFU. A sensitivity analysis for this approach involved a complete records analysis (i.e., including only those people who had complete records for outcome and all variables in the analysis).

#### Estimation of Absolute and Relative Effects.

The 6-year cumulative incidence of each outcome was calculated by dividing the number of events identified at 6-year follow-up by the number of people at risk at the beginning of follow-up; 95% confidence intervals were estimated assuming a Poisson distribution of events. This analysis was done for the population overall, and stratified by key covariates.

Age-adjusted risk ratios of the outcomes (VI and blindness, respectively) were estimated for each covariate using a Poisson regression model with robust error variance to allow for the clustered design and including IPW. For multivariable analysis, an initial model was fitted that included those variables associated with outcome in age-adjusted analysis (Wald *P* value < 0.05). A backward stepwise approach was applied to obtain a final multivariable model, removing variables with *P* > 0.05.

#### Definitions.

Visual acuity: WHO definitions of VI and blindness were used throughout.^20^ Monocular VI was defined as VA < 6/18 (20/60) in either eye. Visual impairment was defined as a VA of <6/18 in the better eye. Monocular blindness was defined as a VA of <3/60 (20/400) in either eye. A person was considered to be blind if the VA in the better eye was <3/60. The definition of VI also includes those who were blind. An estimate of incident monocular and bilateral blindness using the US definition was also calculated. The US definition of monocular blindness is a Snellen acuity of ≤6/60 in either eye and ≤6/60 in the better eye for person blindness.^13^

Diabetes: Diabetes was defined as self-reported in the history, or random glucose ≥ 11.0 mM, or (3) HbA1c ≥ 7.0.

Socioeconomic status: A socioeconomic status (SES) score was developed based on information collected on job, housing conditions, and ownership of material goods and livestock, based on previous work in the same population.^18^

#### Extrapolation of Data.

Estimates of annual cumulative incidence were extrapolated to estimate the number of adults over the age of 50 with incident VI or blindness in Kenya each year. This was calculated by taking the 2015 population estimate from Kenya (Census Bureau of Kenya; Supplementary Table S2) by age category and sex and multiplying this by the age- and sex-specific estimates of annual cumulative incidence.

## Results

At baseline 4381 participants were examined (Fig.). Of these, 2645 (60%) were reidentified at the 6-year follow-up. The reasons for non–follow-up were migration (*n* = 321, 7%), deceased (*n* = 407, 10%), and unknown (*n* = 1008, 23%). Of the baseline participants, 2170 (50%) were reexamined. The large number of unknowns is thought to be due to mass displacement during the postelection violence in Kenya in 2007–2008.^21^ Of the 2170 participants followed up, 2164 (99.7%) had complete data. Those with complete data available were used in the model for missing data and adjustment of estimates. The mean follow-up time of all participants was 5.6 years (SD 0.6) and the median was 5.5 years (inter quartile range, 5.0–6.1), expressed as a “6-year cumulative incidence” from here on. The Supplementary Figure includes the visual status of participants at both time points in the cohort study.

Table 1 provides the baseline characteristics of participants who were reexamined at follow-up and those who were LTFU. In comparison to participants, there was strong evidence that those who had died during follow-up were older, were more likely to be male, and had lower education and SES. Compared with participants seen, those LTFU were less likely to be Kikuyu or Kalenjin speakers, had lower levels of education, were more likely to be from urban areas, and had higher SES.

**Table 1:**
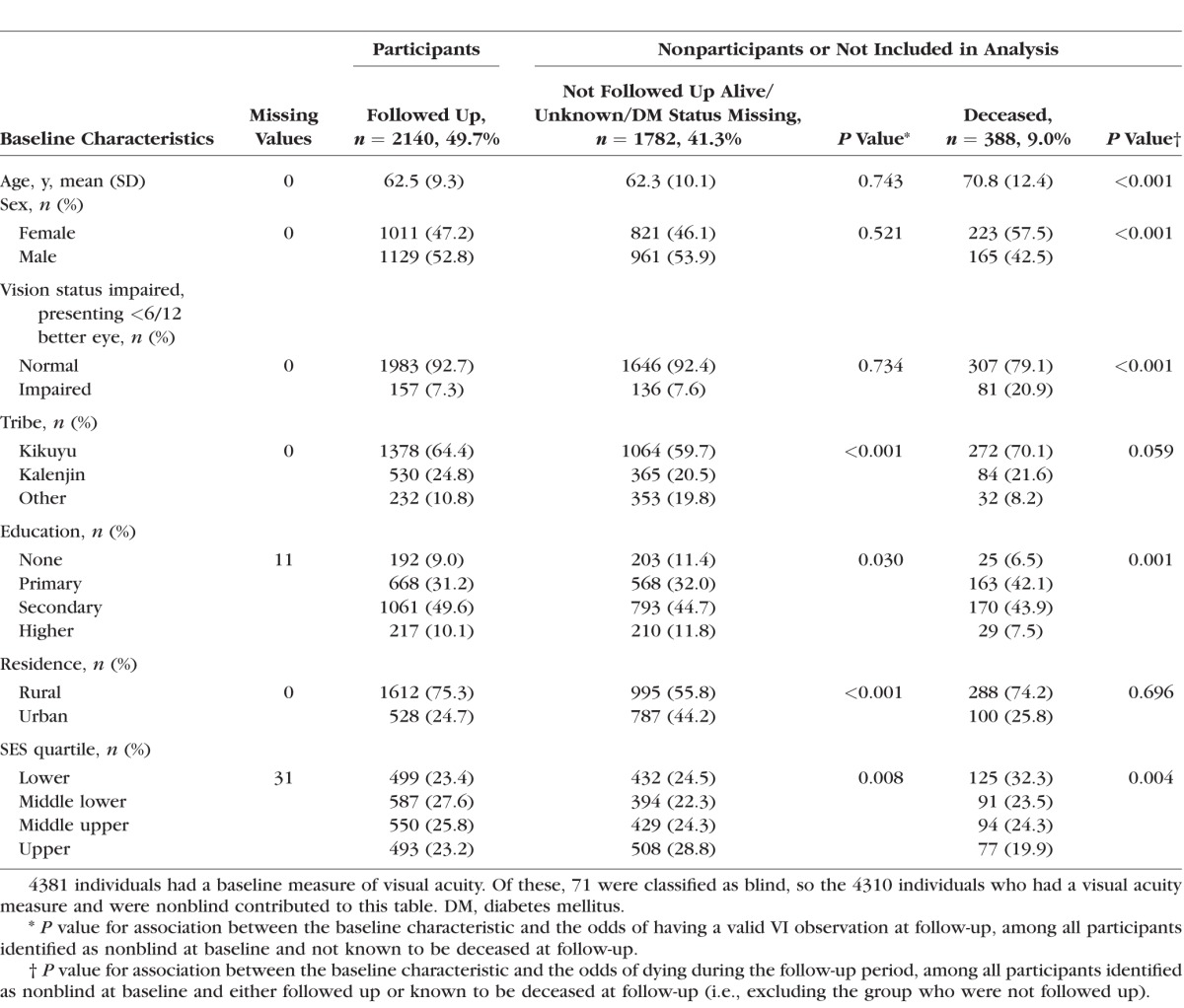
Baseline Characteristics in Nonblind Participants of the Nakuru Eye Disease Cohort Study: Participants and Nonparticipants (*n* = 4310)

In those followed up, the prevalence of VI was higher at follow-up (23%) than at baseline (13%), suggesting an overall shift toward VI in this aging cohort (Table 2). Of the 45 blind at follow-up, the majority were incident cases (*n* = 29, 64%). Eight of 24 blind persons at baseline were no longer blind at follow-up, having received treatment in the interim period; however, only 2 had achieved normal vision (Table 2).

**Table 2:**
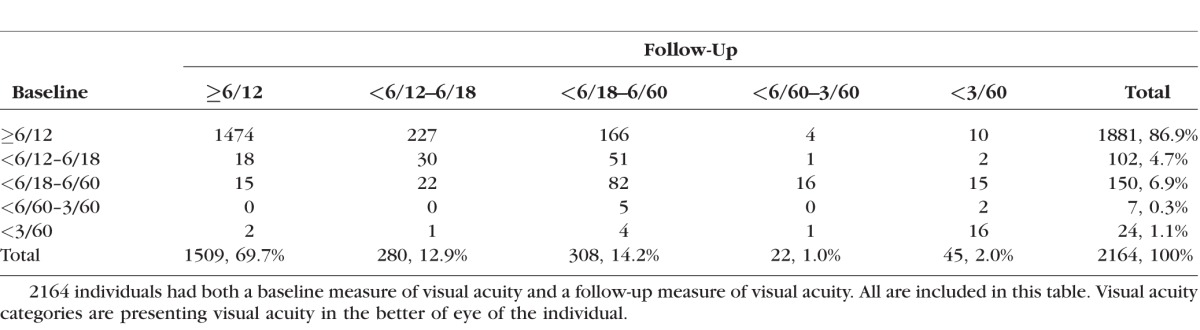
Change in Presenting Visual Acuity Category From Baseline to Follow-Up in Cohort With Visual Acuity Data From Both Time Points (*n* = 2164)

### Incidence of Blindness and Visual Impairment

Of the 2164 participants with complete follow-up data, 24 were blind at baseline and were therefore excluded from the group considered at risk of becoming blind. We analyzed 2140 subjects at risk for incident blindness, of whom 29 participants (1.36%, 95%CI: 0.9–1.9%) were blind at the follow-up visit (Table 3). All subsequent results presented here have been calculated based on the at-risk population and take account of clustering, and also account for missing data via IPW, unless otherwise stated.

**Table 3:**
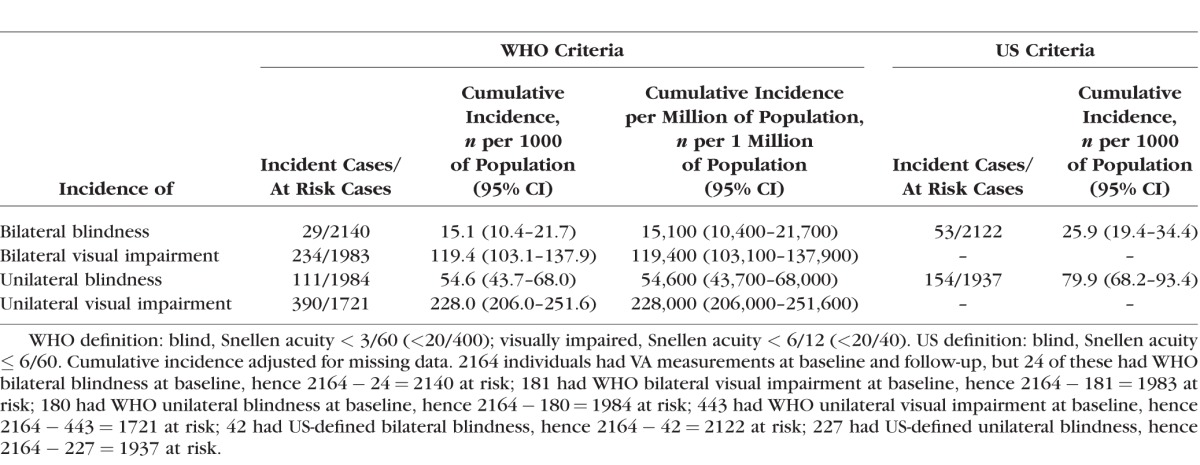
Six-Year Adjusted Cumulative Incidence of Unilateral and Bilateral Visual Impairment by WHO and US Criteria Among the Nakuru Eye Disease Cohort Study Participants

The cumulative incidence, in participants aged 50 years and over, of WHO-defined bilateral and unilateral VI was 119.4/1000 (95%CI 103.1–137.9) and 228.0/1000 (95%CI 206.0–251.6), respectively (Table 3). The cumulative incidence of WHO-defined bilateral and unilateral blindness was 15.1/1000 (95%CI 10.4–21.7) and 54.6/1000 (95%CI 43.7–68.0), respectively. Unweighted estimates using only those participants with complete records of incidence were similar: WHO-defined (Supplementary Table S3) bilateral and unilateral VI was 118.0/1000 (95%CI 102.0–136.2) and 226.6/1000 (95%CI 204.8–250.0), respectively (Supplementary Table S1), with estimates of blindness being slightly lower at 13.6/1000 (95%CI 9.5–19.4).

The cumulative incidence using the US definitions of blindness was estimated to enable comparison with other cohorts, and was higher than for WHO estimates (Table 3). All further analyses are based on WHO definitions.

There was strong evidence of an increase in 6-year cumulative incidence of VI and blindness by age (Table 4). Overall differences in sex across all age categories were not evident; however, a significant difference was found between male and females aged ≥80 years for cumulative incidence of both VI and blindness.

**Table 4:**
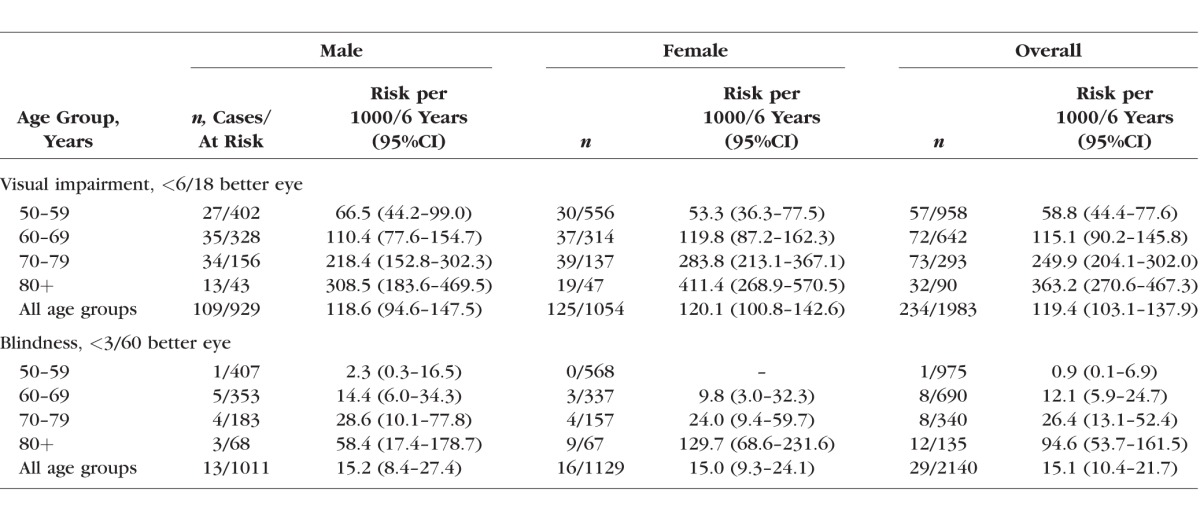
Age- and Sex-Specific 6-Year Adjusted Cumulative Incidence of Visual Impairment and Blindness by WHO Definition Among the Nakuru Eye Disease Cohort Study Participants

Extrapolations based on recent census data were used to calculate the number of individuals aged ≥50, by age and sex, estimated to become visually impaired or blind in Kenya each year (Table 5, Supplementary Table S4). There are an estimated 21 new cases of VI in people aged ≥50 years per 1000 total population per year, of whom 3 (2.7) are blind. Therefore in Kenya we estimate that there are 92,000 new cases of VI per year in people aged ≥50 years, of whom 11,600 are blind, out of a total population of approximately 4.3 million people aged ≥50 (Supplementary Table S5).

**Table 5:**
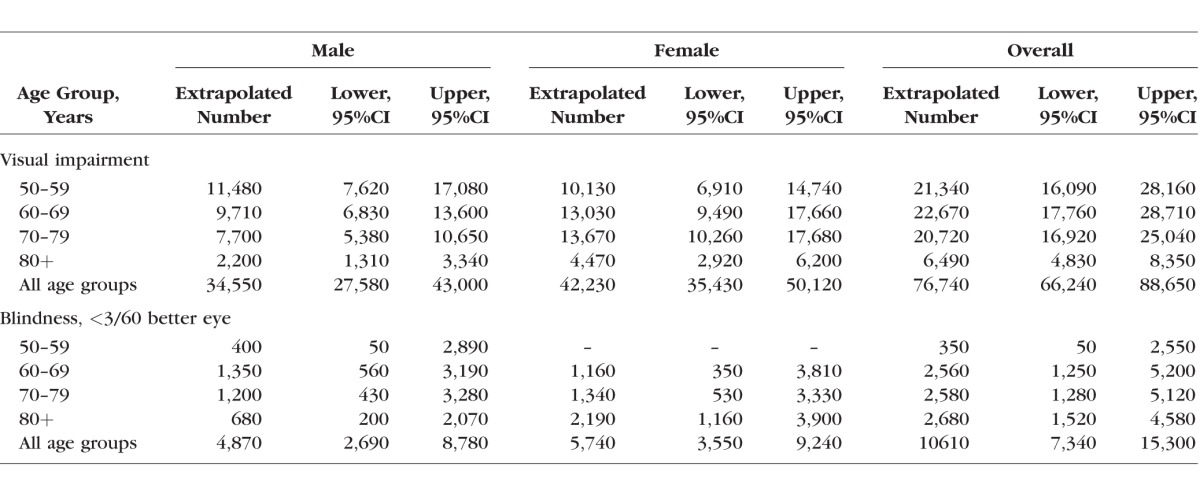
Extrapolated Number of Adults per Year, Aged 50 Years and Over, in Kenya With New Visual Impairment and Blindness Based on Weighted Incidence Data and Estimates of the Population in Kenya by Age Group in 2015

Data from other similar populations indicate that 85% of blindness prevalence is among those aged ≥50 years.^22^ Assuming that the relative incidence of blindness in the under- and over-50s is comparable to the prevalence (i.e., 85% of incidence is also in the over-50s), extrapolating to all ages, we estimate that there are 1.66 new cases of blindness per 1000 per year in all ages in Kenya, approximately 76,000 new cases annually out of a total population of 46 million.

Multivariable analysis for incident bilateral blindness and VI, respectively, showed only diabetes and increasing age to be associated (Table 6). However, low numbers of incident cases of blindness and wide confidence intervals make drawing conclusions limited for this group. There was no evidence of an association with all other risk factors.

**Table 6:**
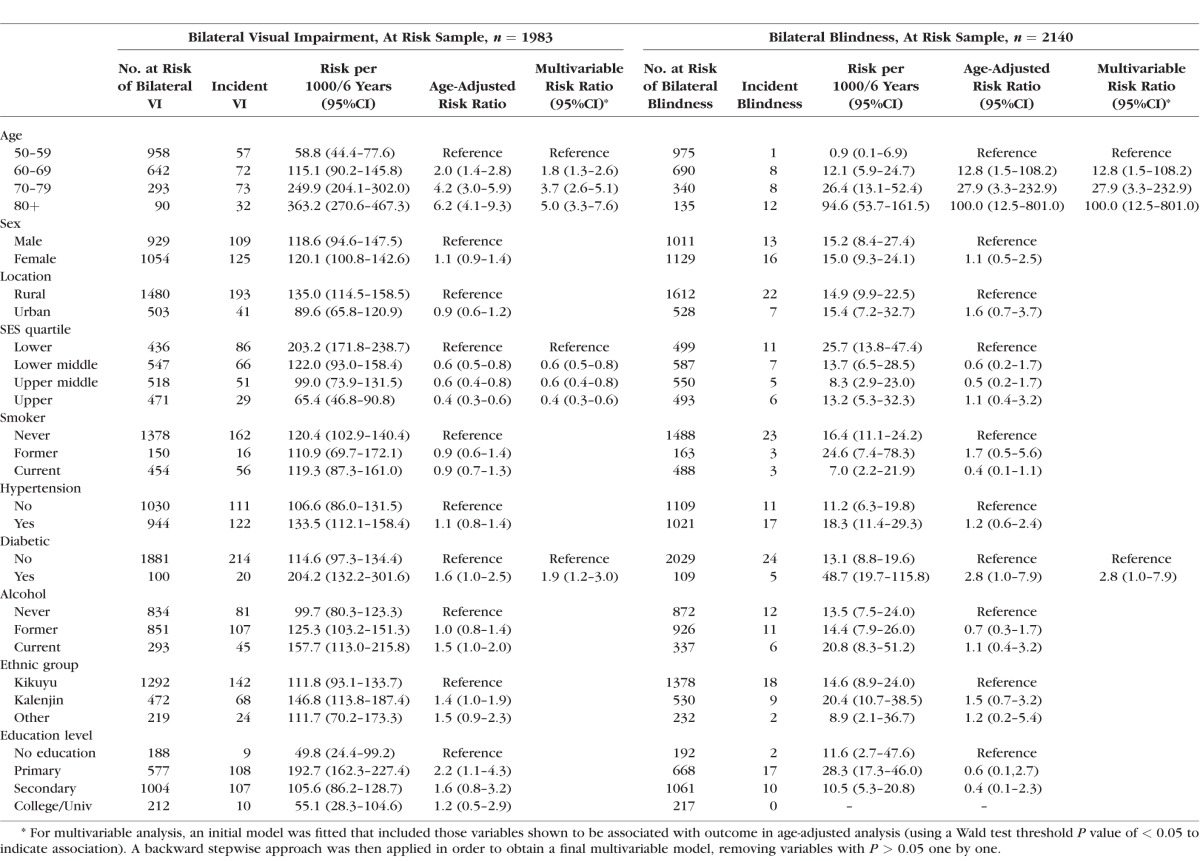
Age-Adjusted and Multivariable Analysis of a Number of Baseline Covariables and Incident Visual Impairment and Blindness in the Nakuru Eye Disease Cohort Study

## Discussion

There are few longitudinal population-based studies describing the incidence of blindness and VI worldwide, and data from sub-Saharan Africa are particularly sparse (Table 7). The data build on our previously reported population-based estimates of prevalence in the same population.^13^

**Table 7:**
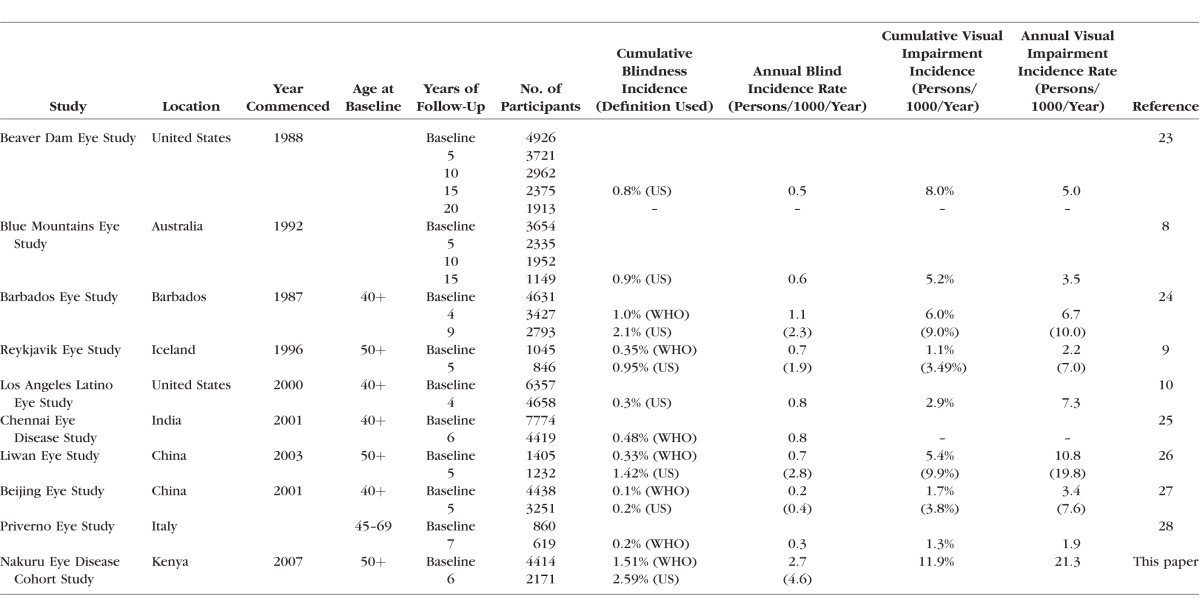
Estimates of Incidence of Blindness From Comparative Population-Based Studies of Eye Disease

We found that the annual incidence of blindness in those aged 50 years and over was 2.2 per 1000 people per year using the WHO definition (VA < 20/400 Snellen in the better-seeing eye) and 4.3 per 1000 for the US definition (VA ≤ 20/200 in the better-seeing eye). The annual incidence of VI (VA < 6/18 Snellen in the better-seeing eye) was 20.9 per 1000 people per year. These estimates are substantially higher annual incidence rates of VI and blindness when compared with other cohort studies (Table 7). It should be noted that comparable studies had varying follow-up periods and thus comparison is made based on annual incidence.

As expected, the incidence of VI and blindness increased significantly with age, as seen in all previous comparable cohort studies. This reflects age-related changes to the crystalline lens and age-related retinal and nerve diseases. The disease-specific incidence rates will be presented and discussed in separate reports.

One previous study from Uganda assessed the incidence of VI and blindness in an African population from a population-based cohort that was established to assess the dynamics of human immunodeficiency virus (HIV) infection through annual censuses and serologic surveys,^29^ and incorporated an assessment of vision at two time points. The sample was a general population cohort and not designed specifically for eye disease, measuring only VA (modified Snellen chart).^30^ Only one case of incident bilateral blindness was reported and 21 cases of incident VI in the study sample (aged 13 and above), providing an age-standardized incidence rate of bilateral VI of 13.2 persons per 1000 persons per year (in a different age group from that presented from this population).^31^ In comparison, this study estimated an incidence of VI at 20.9 persons per 1000 per year.

There are data from comparable population-based studies of eye disease worldwide (Table 7). There are some variations in the age group considered for inclusion, although the majority sampled those 40 or 50 years and above. Most studies presented incident data using the WHO and US definitions of VI or blindness, but some included only one definition, limiting comparability across studies. The incidence of bilateral VI in the Nakuru Eye Disease Cohort Study was found to be higher than anywhere else in the world. The annual incidence rate (persons per 1000 per year) for the majority of studies (eight) was between 0.2 and 0.9 (US classifications) and 0.1 and 0.5 (WHO classifications).^8–10,23,25–28^ Only two studies were higher, at 1.1 (US) and 2.1 (WHO) for the Barbados Eye Study^24^ and 1.2 (US) and 2.4 (WHO) for the Nakuru Eye Disease Cohort Study, respectively.

The high incidence in this study most likely reflects a combination of low access to ophthalmic services and health services in general^32^; there was only one ophthalmologist in the region of the study for a population of approximately 1.6 million people. Other explanations include environmental risk factors including geography, diet, ethnic origin, and ultraviolet light exposure. Other barriers to eye care provision in the region include a low awareness of treatable sight loss, available services that are unaffordable and far away, and fear of treatment.

The data in this study indicated that of 29 new cases of blindness at follow-up, 12 had VA of 6/18 or better, and 17 were worse than 6/18 (see Table 2). Of the 24 who were blind at baseline, 16 were still blind at follow-up. Further analysis will disaggregate incident VI and blindness by cause, enabling further data to support planning (e.g., estimation of need for cataract surgery).

Strengths of the study include the following characteristics: a representative population-based sample in an area of ethnic, socioeconomic, and educational diversity; a large sample size; comprehensive assessment of risk factors; high-quality assessment of vision; and utilization of the same tools at baseline and follow-up. The methodology used to assess ophthalmic disease was consistent with studies performed in well-developed health systems in high-income countries such as the United States^23^ and Australia,^8^ with use of the latest available equipment,^11^ thus making the data highly comparable to those in other population-based cohort studies of eye disease.

The major limitation of this study was low participation rate (50%) at 6 years; however, having the baseline characteristics of nonparticipants is a strength that enabled weighting to ensure better estimates of cumulative incidence. This loss to follow-up may have led to an under- or overestimation of incident VI and blindness, depending on the general characteristics of the nonrespondents. The predominant risk factor for incident VI or blindness was age; and given that this was closely matched between participants and nonparticipants (62.7 years [SD 9.4] and 62.5 years [SD 10.4], respectively), the estimates are likely to be an acceptable reflection. This assessment is further supported in that minimal changes were apparent after adjusting estimates for missing data (Supplementary Table S1).

Reasons for the low participation included ethnic violence and displacement of large numbers of people in the study sample area. Postelection violence in 2007 and 2008 led to up to 600,000 people being internally displaced and 1300 fatalities.^21^ In a number of study clusters, entire ethnic groups present at baseline were no longer available or traceable. Great efforts were made to locate individuals. Further limitations include restricting the inclusion criteria at baseline to those 50 years and above, thereby reducing the generalizability of our results to the entire population. This is, however, comparable to the majority of population-based studies of eye disease that restrict inclusion to 40 or 50 years and above (Table 7). Furthermore, the majority of prevalent and incident vision loss is in this age group, making the sample size feasible.^22^ The definition of blindness and VI in this study did not include peripheral vision loss and was based solely on presenting central logMAR VA. This potentially underestimates the incident VI and blindness (particularly from glaucoma) when compared to studies that include these criteria.

Our results suggest that there are 86,000 new cases of VI in people aged ≥50 years per year in Kenya, of whom 8100 are blind. Recent estimates suggest that there are 86 ophthalmologists in Kenya^33^ for a population of approximately 45 million, with the majority (50%) being based in the capital city of Nairobi. This leaves 92% of the population (approximately 40 million people) being served by 43 ophthalmologists. Overall, Kenya is better resourced than many other African countries in terms of human resources, despite still being well below recommended targets.^34^ Continued effort to strengthen the eye health system is necessary to support the growing unmet need of this aging and growing population.

In conclusion, the incidence of VI and blindness in this adult Kenyan population was considerably higher than in comparable studies worldwide. Further analyses on the causes of incident blindness will help in setting priorities for preventing avoidable blindness in this population.

**Figure:**
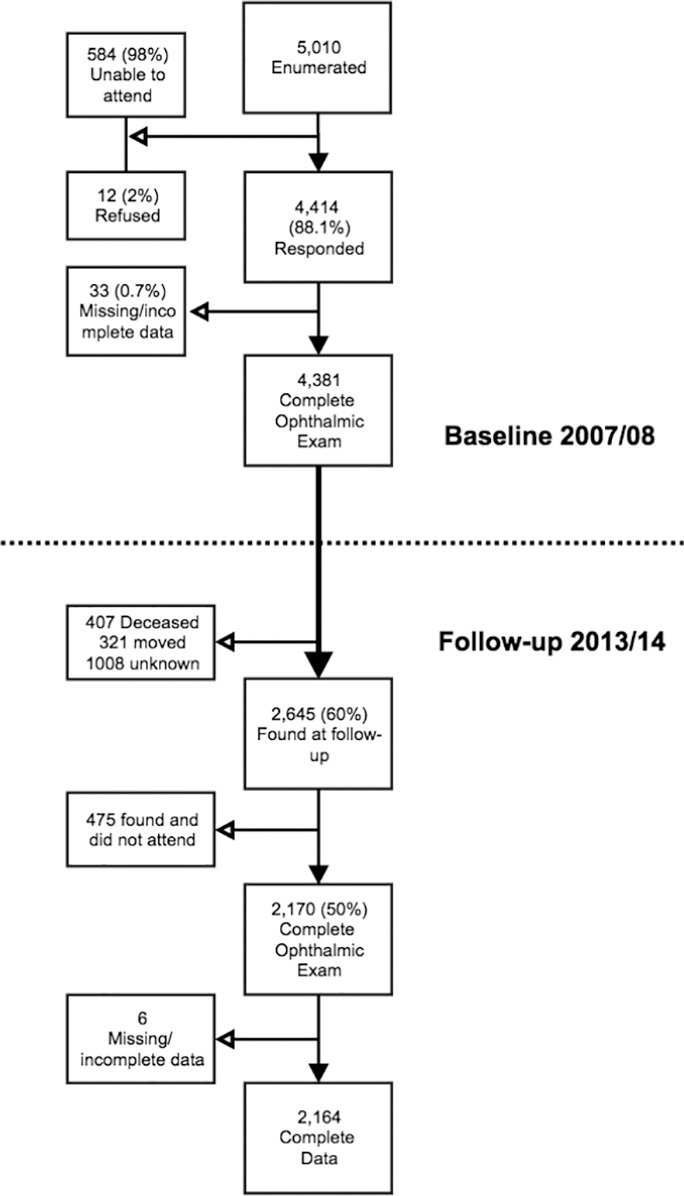
Flowchart of participants and nonparticipants.

## Supplementary Material

Supplement 1

Supplement 2
