# Supplementary figures and images for: Six-Year Incidence of Blindness and Visual Impairment in Kenya: The Nakuru Eye Disease Cohort Study
Source: Invest Ophthalmol Vis Sci. 2016 Nov;57(14):5974–83. doi: 10.1167/iovs.16-19835 (PMC5102568; doi:10.1167/iovs.16-19835)

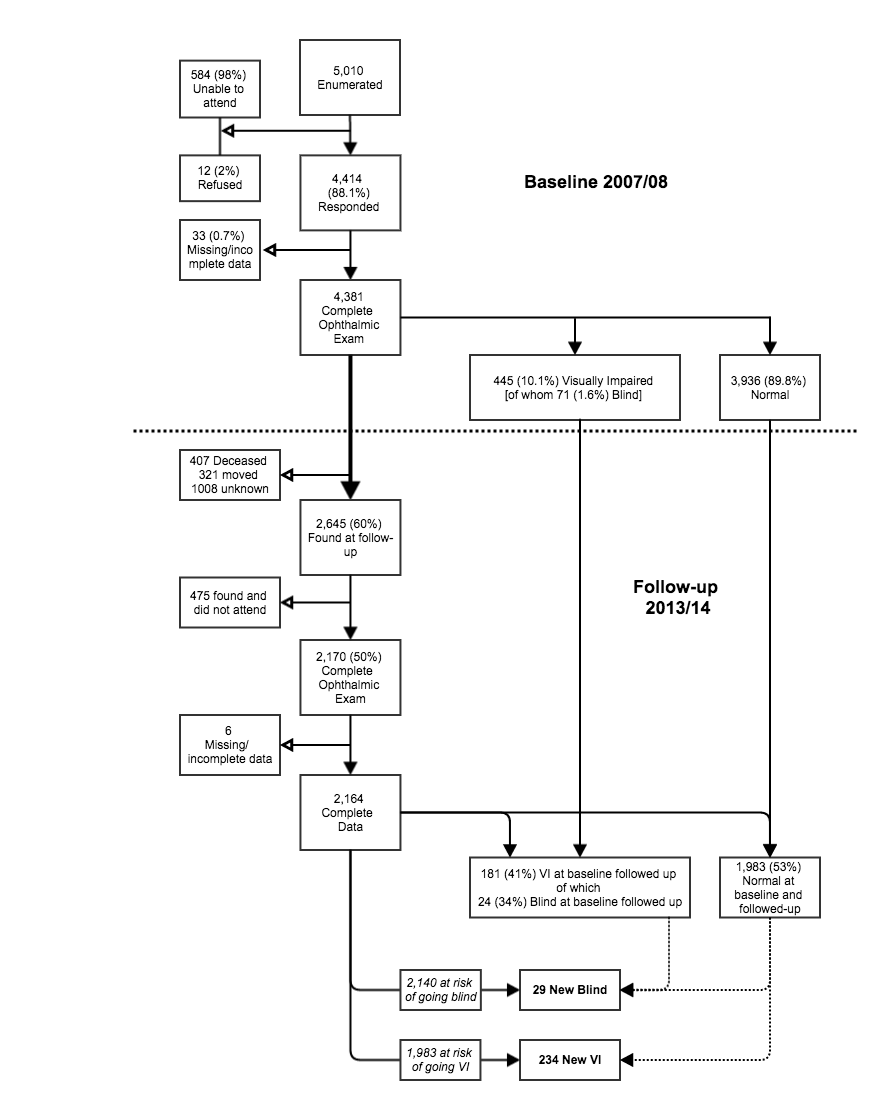

Supplement: Supplement 1 [file iovs-57-11-36_s01.png]
