# Supplementary material for: Six-Year Incidence of Blindness and Visual Impairment in Kenya: The Nakuru Eye Disease Cohort Study
Source: Invest Ophthalmol Vis Sci. 2016 Nov;57(14):5974–83. doi: 10.1167/iovs.16-19835 (PMC5102568; doi:10.1167/iovs.16-19835)
Supplement: Supplement 2 [file iovs-57-11-36_s02.pdf]

Supplementary Material

Data cleaning and management

All raw data collected during the fieldwork was imported from Excel into STATA and the variables and categories of the collected variables were labelled appropriately, before checking for missing data and likely input errors. Any issues identified were discussed within the study team, in order to identify whether the issue was expected based upon the study design and data collection approach used in the field. Unexpected issues were resolved by checking the source data. Outcome variables were then generated, based on the collected field data. These included blindness and visual impairment (at follow-up) outcome variables per person and per eye (i.e. monocular) according to the WHO (for both blind and visual impairment) and the U.S. (for blind only) standards (see Supplementary Table S1).

Co-variate (recorded at baseline) variables were then set up for age, gender, education, diabetes, socioeconomic status (SES) and hypertension. A categorical age variable was created (10 year age categories from 50 onwards). Education was defined as none, primary, secondary and higher. SES included lower, middle lower, middle upper and upper quartile categories. All other co-variates were setup as binary variables (i.e. male/female or no/yes, with male and no as baseline categories). A dedicated cohort analysis dataset was then created that contained the unique study ID for each individual, the village of residence (which defined the cluster in subsequent analysis), the individual’s follow up status (i.e. participant vs non-participant), whether the person died or not, the outcome variables and the covariates.

Table S1. Baseline Characteristics in Nakuru Eye Disease Cohort Study: Participants and Non-participants

		**Participants**	**Non-participants**			
**Baseline Characteristics**		*(n=2,171, 49.2%)*	*Alive/Unknown n=1,834, (41.5%)*	*p-value*	*Deceased (n=409, 9.3%)*	*p-value*
Age (yrs), mean +/- SD		62.7 (9.4)	62.5 (10.4)	0.50	71.6 (12.8)	<0.001
Male % (n)		47.3% (1,026)	46.4% (851)	0.59	57.7% (236)	<0.001
Vision status	Normal (≥6/12 both eyes)	91.6% (1,988)	91.0% (1,641)	0.47	75.4 % (307)	<0.001
	Impaired (<6/12 better eye)	8.4% (182)	9.0% (163)		24.6% (100)	
Tribe % (n)	Kikuyu	64.2% (1,393)	59.1% (1,084)	<0.001	69.2% (283)	0.10
	Kalenjin	25.1% (544)	20.6% (378)		22.7%(93)	
	Other	10.8% (234)	20.3% (372)		8.1% (33)	
Education % (n) [a]	None	8.9% (193)	11.1% (204)	<0.001	6.4% (26)	<0.001
	Primary	31.7% (689)	32.0% (586)		43.8% (179)	
	Secondary	49.3% (1,070)	43.9% (805)		42.5% (174)	
	Higher	10.0% (217)	11.7% (215)		7.1% (29)	
Urban % (n)		24.5% (532)	44.1% (808)	<0.001	25.9% (106)	0.54
SES Quartile % (n)[b]	Lower	23.8% (517)	23.9% (439)	<0.001	33.3% (136)	0.002
	Middle lower	27.2% (591)	22.0% (404)		23.5% (96)	
	Middle upper	25.7% (557)	23.9% (438)		23.7% (97)	
	Upper	22.8% (495)	28.2% (517)		19.3% (79)	

P values represent the difference between participants and non-participants (deceased and

alive/unknown), results of a chi-squared test of overall association of the variable.

a. Missing data on 27 participants, b. missing data on 48 participants

Note 24 participants followed up were blind at baseline, therefore n= 2,160 – 24 = 2,136

**Table S2. Estimates of Population of Kenya in 2015**			
**Age**	**Both Sexes Population**	**Male Population**	**Female Population**
All ages	45,925,301	22,907,500	23,017,801
50-59	2,231,660	1,061,476	1,170,184
60-69	1,278,899	565,224	713,675
70-79	594,118	258,604	335,514
80+	187,131	78,468	108,663
50+	4,291,808	1,963,772	2,328,036

Table S3. Six-Year unweighted Cumulative Incidence of Unilateral and Bilateral Visual Impairment by World Health Organization and United States Criteria among the Nakuru Eye Disease Cohort Study Participants.

	WHO criteria			US criteria	
Incidence of	Incident cases / At risk cases	Cumulative incidence (n / per 1000 of population, 95% CI)	Cumulative incidence per million of population (n / per 1m of population, 95% CI)	Incident cases / At risk cases	Cumulative incidence (n / per 1000 of population, 95% CI)
Bilateral blindness	29 / 2140	13.6(9.5,19.4)	13,600 (9,500-19,400)	53 / 2122	25.0(18.9,33.0)
Bilateral Visual Impairment	234 / 1983	118.0(102.0,136.2)	118,000 (102,000-136,200)	-	-
Unilateral blindness	111 / 1984	55.9(45.3,68.8)	55,900 (45,300-68,800)	154 / 1937	79.5(68.2,92.4)
Unilateral Visual Impairment	390 / 1721	226.6(204.8,250.0)	226,600 (204,800-250,000)	-	-

Table S4. Age-Gender–Specific 6-Year unweighted cumulative incidence of Visual Impairment and Blindness by World Health Organization definition among the Nakuru

	Male		Female		Overall	
Age Group (years)	n (Cases / at risk)	Risk per 1,000/6yrs (95%CI)	n	Risk per 1,000/6yrs (95%CI)	n	Risk per 1,000/6yrs (95%CI)
Visual Impairment (<6/18 better eye)						
50-59	27 / 402	67.2(47.5,94.1)	30 / 556	54.0(37.7,76.6)	57 / 958	59.5(46.1,76.4)
60-69	35 / 328	106.7(74.9,149.9)	37 / 314	117.8(85.4,160.5)	72 / 642	112.1(87.2,143.1)
70-79	34 / 156	217.9(151.6,303.0)	39 / 137	284.7(212.2,370.3)	73 / 293	249.1(202.8,302.0)
80+	13 / 43	302.3(183.8,454.8)	19 / 47	404.3(264.1,562.0)	32 / 90	355.6(264.7,458.2)
All age groups	109 / 929	117.3(94.3,145.0)	125 / 1054	118.6(99.3,141.1)	234 / 1983	118.0(102.0,136.2)
Blindness (<3/60 better eye)						
50-59	1 / 407	2.5(0.3,17.7)	0 / 568	-	1 / 975	1.0(0.1,7.5)
60-69	5 / 353	14.2(6.0,33.2)	3 / 337	8.9(2.9,27.4)	8 / 690	11.6(5.8,23.0)
70-79	4 / 183	21.9(8.1,57.6)	4 / 157	25.5(10.1,62.7)	8 / 340	23.5(12.1,45.2)
80+	3 / 68	44.1(13.7,133.0)	9 / 67	134.3(73.1,233.9)	12 / 135	88.9(50.2,152.6)
All age groups	13 / 1011	12.9(7.3,22.4)	16 / 1129	14.2(8.9,22.5)	29 / 2140	13.6(9.5,19.4)

Table S5. Extrapolated number of new adults per year, aged 50 years and over in Kenya with visual impairment and blindness based on unweighted incidence data and estimates of the population in Kenya by age group in 2015.

	**Male**			**Female**			**Overall**		
Age Group (years)	Extrapolated number	Lower (95%CI)	Upper (95%CI)	Extrapolated number	Lower (95%CI)	Upper (95%CI)	Extrapolate d number	Lower (95%CI)	Upper (95%CI)
Visual Impairment									
50-59	11590	8200	16230	10260	7180	14560	21580	16740	27710
60-69	9380	6580	13180	12820	9290	17450	22090	17180	28180
70-79	7680	5340	10680	13710	10220	17840	20660	16820	25040
80+	2150	1310	3240	4390	2870	6110	6350	4730	8190
All age groups	34190	27500	42260	41680	34880	49600	75830	65530	87510
Blindness (<3/60 better eye)									
50-59	430	60	3110	#VALUE!	#VALUE!	#VALUE!	380	50	2760
60-69	1320	560	3100	1050	340	3230	2450	1230	4860
70-79	920	340	2430	1420	560	3500	2300	1180	4420
80+	510	160	1540	2260	1230	3940	2520	1420	4330
All age groups	4120	2350	7190	5430	3410	8630	9540	6660	13640
